# Physical and Biochemical Properties of Chan (*Hyptis suaveolens* (L.) Poit) Seeds Grown in Different Locations

**DOI:** 10.1002/fsn3.71490

**Published:** 2026-03-13

**Authors:** Juan Alfredo Salazar‐Montoya, María Dolores Díaz‐Cervantes, Emma Gloria Ramos‐Ramírez

**Affiliations:** ^1^ Biotechnology and Bioengineering Department Cinvestav México City Mexico

**Keywords:** atomic force microscopy, chan seed, fatty acids, physical properties, scanning electron microscopy

## Abstract

The main goal of this work was to establish the differences in the physical properties and nutritional quality of chan seeds ( *Hyptis suaveolens* (L.) Poit) as well as to determine their influence on the structural characteristics. In this study, three sites along the eastern coast of Mexico were selected for sample collection since they are the areas in which chan is produced. Its protein content is comparable to pseudocereal (17.92%–19.63%). For their part, in fats (17.81%–18.38%), ῳ‐6 (74.67%–78.19%) and ῳ‐9 (9.31%–11.32%) acids mainly predominate. The high crude fiber content (18.79%–20.85%) allows the prebiotic potential of the seeds to be considered. Physical properties showed variations between crops: length (3.275–3.559 mm), width (2.443–2.635 mm), perimeter (10.139–12.202 mm), and weight (5.161–5.651 g/1000 seeds), occupying a volume of 4.913–5.174 mm^3^. The true density ranged between 1.056 and 1.088 g/cm^3^; bulk density was 0.616–0.621 g/cm^3^; the static (28.31°–29.73°) and dynamic (35.21°–40.58°) angle of repose also varied between crops. The surface, thickness, arithmetic and geometric diameter, percentage of sphericity, and porosity were determined and the volume of PDI was calculated, which ranged between 5.649 and 6.226 mm^3^. Pearson correlation coefficient determined the relationship between the properties studied, with *n* = 120 determinations per parameter (*p* < 0.05). In the microstructure of the exocarp of the seeds, there is an areolar zone of globular cells and a predominant zone (mixocarpy) which is characterized by being formed by intermingled mucilaginous and non‐mucilaginous cells of isodiametric and angular shape. This study provides knowledge for modernizing sowing and post‐harvest for intensive cultivation. This study contributes essential knowledge for the modernization of sowing and post harvest practices in intensive cultivation systems. It also provides the basis for establishing standardized procedures and designing equipment for the efficient handling, processing, storage, and distribution of agricultural products.

## Graphical abstract

Physical properties of the chan seeds were determined and correlated with each other. The Pearson correlation coefficient determined the relationship between the parameters studied. Physicochemical properties of chan seeds were evaluated, determining proteins, fatty acids (linoleic acid oleic acid), and crude fiber. The mixocarpy of the chan seeds was determined by SEM, which is the predominant micromorphology in the seeds, formed by isodiametric and angular cells. Physical parameters help to develop better post‐harvest, industrial, and distribution processes.

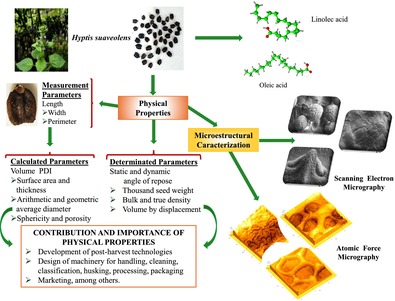

## 1 Introduction

Humanity faces grave problems due to demographic growth of 8000 million in November 2022. It is estimated that the world population will reach around 9250 million by 2030 (United Nations Department of Economic and Social Affairs 2022). Climate change can cause droughts and increased temperatures, affecting crop areas, in addition to dependence on a reduced number of crops for food (de la Cruz Torres et al. 2013; Reza and Sabau 2022). This implies the great challenge of satisfying these nutritional needs that can be covered with the consumption of seeds. Globally, approximately 70% of food comes mainly from cereals such as wheat, corn, rice, and, to a lesser extent, legume seeds. These provide different carbohydrates, lipids, and proteins for man (de la Cruz Torres et al. 2013). Therefore, the need arises for new agricultural strategies that promote the cultivation of underutilized species with high nutritional value and functional properties.

The chan ( *Hyptis suaveolens* (L.) Poit) belongs to the *Lamiaceae* family. It is native to the tropical region of America, and it is considered a worldwide weed (Tang et al. 2019). It is distributed in more than 50 countries on all continents (Li et al. 2020). In Mexico, this plant was widely cultivated in pre‐Hispanic times for its edible seeds and is currently considered a “pseudocereal” (Sanchez‐Aguirre et al. 2020). Nowadays, chan is cultivated in seasonal tropical climate zones that cover more than 31.3% of the country's forest area and are distributed mainly along the Pacific coast (García‐Oliva et al. 2002), which includes various regions, primarily Colima, Jalisco, and Nayarit. In Colima state, three varieties are located: the wild, the weed or hybrid, and the domesticated variety (Vergara‐Santana et al. 2005).

In various countries, different parts of the plant have been used in traditional medicine to treat gastrointestinal, respiratory, rheumatoid conditions, and fever (Sanchez‐Aguirre et al. 2020; Tang et al. 2019). However, *Hyptis suaveolens* , despite being a plant with high medicinal, insecticidal, and antioxidant value, have been little studied. This plant is notable for its diverse profile of phytochemical compounds, including sterols, flavonoids, and phenolic constituents, among others. It also contains terpenes and ursolic acid, both of which have been associated with antiviral activity against SARS CoV2 (Aguele et al. 2023; Mishra et al. 2021). Chan seeds contain proteins with high content of aromatic and branched chain amino acids. They are a source of essential minerals in human nutrition, such as calcium, potassium, magnesium, iron, zinc, copper, and sodium (Bachheti et al. 2015; Umedum Ngozi et al. 2014), and fatty acids predominantly polyunsaturated fatty acids (PUFAs) and monounsaturated fatty acids (MUFAs) (Mueller et al. 2017). This combination of fatty acids allows chan seed oil to be of high nutritional quality, due to having a greater amount of hypocholesterolemic fatty acids, due to its reduced degree of thrombogenicity, and low atherogenicity index (Díaz‐Cervantes et al. 2023). In addition, the seed produces an extracellular polysaccharide with a high water retention capacity, which is due to its gelling, emulsifying, and stabilizing capacity. These functional properties have potential as a prebiotic and are suitable for various industrial applications (Morales‐Tovar et al. 2020). The importance of this plant is widely recognized, and there is growing short‐term interest in developing its intensive cultivation. This interest is closely linked to the potential for industrialization, as its components have nutraceutical and functional properties (Ahoton et al. 2010; de Mesquita Arruda et al. 2018). This incipient development implies challenges to develop technologies and machinery that allow the management, distribution, processing, and commercialization of seeds in the postharvest stage, which implies challenges in operations. In this context, the measurement of seed physical properties provides essential information for the technification of sowing processes, the implementation of post harvest methods, the establishment of operating parameters, and the design of equipment for harvesting, cleaning, classification, fractioning, dehulling, crushing, packaging, and distribution (Adubofuor et al. 2021; Kaliniewicz and Choszcz 2021; Soyoye et al. 2018).

The physical properties of various seeds have previously been evaluated. Porras‐Loaiza et al. (2014) studied the morphological features of chia seeds from different regions of Mexico and their relationship between them. There are also reports of these features and their comparison between varieties of orange seeds; in addition, with seeds of nutritional importance, such as the pseudocereals amaranth, quinoa, and buckwheat (Rosa et al. 2020); peanut seed (Chukwu et al. 2018; Reddy and Mathew 2021); Mexican pinion (Pensamiento‐Niño et al. 2019); and oilseeds such as sesame, soybean, flaxseed, and sunflower (Araujo et al. 2018; Krajewska et al. 2016; Viveiros de Oliveira et al. 2021).

The physical properties of seeds constitute a useful analytical tool, as they enable the correlation of parameters that determine seed size, shape, and mass, among other traits. These properties also facilitate the assessment of variability among cultivars originating from different geographical regions (Loukhmas et al. 2021), the detection of genetic variation within populations, and the evaluation of their responses to environmental factors. From an agronomic point of view, the study of these physical properties can provide information on the physiological quality of the seed (Dutra et al. 2019; Vilcatoma‐Medina et al. 2017).

It is important to note that some authors refer to the physical properties and measurements of seeds as biometric parameters, as they allow for the evaluation of their genetic variability, quality, and germination and development potential. These parameters can be evaluated both within a single species and between different species. Its determination focuses on population variability, considering aspects such as physical dimensions and morphometry and internal composition (e.g., endocarp, embryo size, and weight). It also considers seedling characteristics, such as root length and vigor index, among others (Camillo et al. 2014; da Costa and Trovão 2020; Oliveira et al. 2024). The present study focused on determining the physical properties and morphometry of seeds. Therefore, the main goal of this study was to determine and calculate the physical properties of chan seeds from different crops and to know about their fatty acid profile and microstructure.

## 2 Materials and Methods

### 2.1 Materials

In this study, Chan ( *Hyptis suaveolens* (L.) Poit) seeds from three locations from the central‐western Pacific of Mexico were used. Chan seeds from Colima were donated by Technological Institute of Roque; the other samples were acquired in the municipal market of Puerto Vallarta, Jalisco, and the market “La Abejita” in Bucerías, Nayarit.

### 2.2 Physical Properties

#### 2.2.1 Length, Width, and Perimeter

The dimensions of length, width, and perimeter were determined by the image digitalization method (Varnamkhasti et al. 2007); with a scanner (Deskjet 3510 e‐All‐in‐One series; HP, USA). One Hundred randomly selected seeds were placed in the scanner. The images were processed using Image‐ProPlus software ver. 5.1 for Windows (Media Cybernetics Inc. USA).

#### 2.2.2 Thousand Seed Weight

Groups of 100 seeds were randomly selected (Araujo et al. 2018; Porras‐Loaiza et al. 2014) that were weighed on an analytical balance (Ohaus Analytical Plus, USA). Subsequently, this weight was extrapolated to 1000 seeds, according to the following Equation (1):

(1) $$\text{Weight of}\;1000\;\text{seed}=\sum_{n=1}^{n=8}n\times 1.25$$

#### 2.2.3 Volume by Displacement

The determination was made by the displacement method, using toluene, for having low surface tension and not absorbing the seed. A known volume of toluene was measured in a test tube at 25°C, and a certain number of seeds was submerged. The difference between the initial volume and final volume is the volume occupied by the seed (Agrawal et al. 2015; Mansouri et al. 2017).

#### 2.2.4 True and Bulk Density

The determinations were made on an analytical balance (Ohaus Analytical Plus, USA) weighing a seeds sample. The true density was estimated as the ratio of the mass of the sample as a function of its displacement volume, Equation (2). The bulk density includes the total volume of the seeds and the interstitial spaces between them in the true density; empty spaces are excluded (Rojas Barahona 2010). For bulk density, the volume occupied by the sample was determined in a test tube, according to Equation (3) (Agrawal et al. 2015; Bhattacharya et al. 1993; Mansouri et al. 2017; Varnamkhasti et al. 2007):

(2) $$\rho_{t}=\frac{W_{s}}{V_{d}-V_{i}}$$

(3) $$\;\rho_{b}=\frac{W_{s}}{V_{s}}$$

where $\rho_{t}$= true density (g/cm^3^), $\rho_{b}$= bulk density (g/cm^3^), *W*_*s*_ = Sample weight (g), *V*_*d*_ = displacement volume (cm^3^), *V*_*i*_ = initial toluene volume (cm^3^), *V*_*s*_ = sample volume (cm^3^).

#### 2.2.5 Static and Dynamic Angle of Repose

The static and dynamic angles of repose were measured with an apparatus consisting of a wooden box of 60 mm by 60 mm by 35 mm high and two plates, one fixed and another adjustable. The seeds are dropped into the container box from a height of 20 cm to fill and level it. The adjustable plate was gradually tilted to the position where the seeds reached a slope before starting to move. At that point, the angle was measured on a digital conveyor. This point corresponds to the static angle of repose or filling angle, which was measured on a digital conveyor. Afterward, the plate continues to be moved upward until the seeds begin to move. This position corresponds to the dynamic angle of repose or emptying angle and is read on a digital conveyor (Agrawal et al. 2015; Sahin and Sumnu 2006; Tabatabaeefar 2003; Varnamkhasti et al. 2007).

### 2.3 Calculated Physical Parameters

Some determined physical properties were used to calculate others:

#### 2.3.1 Surface Area and Thickness

It was determined using orthogonal dimensions, applying the following equation (Varnamkhasti et al. 2007):

(4) $$A_{s}=\frac{\mathrm{\pi W}L^{2}}{2L-W}$$

where *A*_*s*_ = surface area (mm^2^), *W* = width (mm), *L* = length (mm).

The unknown thickness of the nth seed is predicted (Cleva et al. 2013) using the following equations:

(5) $$E_{n}=kW_{n}$$

(6) $$\;k=\frac{6V_{\exp}}{\pi \sum_{n=1}^{N}L_{n}W_{n}^{2}}$$

where *E*_*n*_ = Thickness of n‐seed (mm), *k* = Adjustment parameter, *W*_*n*_ = Width of n‐seed (mm), *V*_exp_ = Volume of the sample by the toluene displacement method (mm^3^), *L*_*n*_ = Length of n‐seed (mm).

#### 2.3.2 Arithmetic and Geometric Average Diameter

They were calculated according to the following equations (Agrawal et al. 2015; Coşkuner and Karababa 2007; Sahin and Sumnu 2006; Tabatabaeefar 2003; Varnamkhasti et al. 2007; Vilche et al. 2003):

(7) $$D_{a}=\frac{\left(L+W+T\right)}{3}$$

(8) $$\;D_{g}={\left(\mathrm{LWT}\right)}^{\frac{1}{3}}$$

where *D*_*a*_ = arithmetic average diameter (mm), *D*_*g*_ = geometric average diameter (mm), *L* = length (mm), *W* = width (mm), *T* = thickness (mm).

#### 2.3.3 Sphericity and Porosity

Sphericity is the characteristic shape of a solid object relative to the surface area of a sphere having the same volume as the surface of the seed (Agrawal et al. 2015; Bhattacharya et al. 1993; Kumar and Bhattacharya 2008; Sahin and Sumnu 2006; Tabatabaeefar 2003; Varnamkhasti et al. 2007). It was determined according to Equation (9):

(9) $$\varphi =\frac{D_{g}}{L}\times 100$$

where φ = sphericity (%), *D*_*g*_ = geometric diameter (mm), *L* = length (mm).

The porosity is the fraction of air space between seeds relative to a unit of volume of the same seeds (Agrawal et al. 2015; Bhattacharya et al. 1993; Coşkuner and Karababa 2007; Tabatabaeefar 2003; Varnamkhasti et al. 2007; Vilche et al. 2003). It was determined by the equation:

(10) $$\varepsilon =\frac{\left(\rho_{t}-\rho_{b}\right)}{\rho_{t}}\times 100$$

where ε = porosity (%), *ρ*_*t*_ = true density (g/cm^3^), *ρ*_*b*_ = bulk density (g/cm^3^).

#### 2.3.4 Volume Determined by Procedure Digitalization of Images (PDI)

The model of Varnamkhasti et al. (2007) was used to determine the volume of the seed by correlating its length, width, and thickness according to Equation (11):

(11) $$V_{\mathrm{PDI}}=0.25\left[\left(\frac{\pi }{6}\right)L{\left(W+T\right)}^{2}\right]$$

where *V*_PDI_ = volume by procedure digitalization of images (mm^3^), *L* = length (mm), *W* = width (mm), *T* = thickness (mm).

### 2.4 Proximate Analysis

The analysis of moisture, ash, protein, fat, and crude fiber of the chan seeds was determined by methods of the Association of Official Analytical Chemists (Association of Official Analytical Chemists 2007).

### 2.5 Fatty Acid Composition of Chan Seeds

Chan seed oil was obtained by extraction with petroleum ether in a multi‐unit extractor soxhlet equipment (Lab‐line Instruments Inc. USA) according to the method described by Bachheti et al. (2015). The fatty acids in the oil of the chan seeds were determined by gas chromatography with a flame ionization detector, with the previous formation of the fatty acid methyl esters (Metcalfe et al. 1966) and a ZB‐Wax column 30 m × 0.25 mm × 0.25 μm.

The temperature of the detector was 250°C, the injector 230°C, and the column 160°C for 3 min, with the nitrogen flow of 9 psi. Quantification was done by normalization and identification with acid standards.

### 2.6 Microstructural Characterization

#### 2.6.1 Scanning Electron Microscopy

The structural characterization was carried out through a seed surface scan using a high‐resolution scanning electron microscope (FE HRSEM Auriga 3916, Japan), which consists of a GENIMI Schottky field emission column that works in the range from 0.1 keV to 30 keV and reaches a resolution of 1.0 nm at 15 kV. Each sample adheres to a tape with conductive glue. This equipment allows us to observe low moisture samples without any treatment (Miyazaki et al. 2012).

#### 2.6.2 Atomic Force Microscopy

Topographic characterization was performed using an atomic force microscope (Autoprobe CP Research, Thermo Microscopes group Venco, USA). A 10–15 μm probe, tip length 450 ± 10 μm, tip width 50 ± 7.5 μm, resonance frequency 6–21 KHz, and constant force of 0.02–0.77 N/m were used. It was used in contact mode with a force of 6 nN, gain of 0.5, and curvature radius of 20 nm. The seeds were adhered with carbon tape and different areas of the surface were selected; in each of them, sweeps were made to different areas.

### 2.7 Statistical Analysis

The results were expressed as the average and standard deviation. A one‐way analysis of variance (ANOVA) with a 95% confidence level (*p* < 0.05) was used to determine significant differences between samples, using the Tukey method. The Pearson correlation coefficient was determined to assess the magnitude of the linear relationship between pairs of physical properties and of these with the type of fatty acids present. Minitab software ver.17 (Minitab Inc., USA) was used for statistical analysis.

## 3 Results and Discussion

### 3.1 Physical Properties

There are no reports in the literature about the physical properties of chan seeds. The parameters studied are presented in Table 1, and those calculated parameters from the first in Table 2. The values correspond to the means, standard deviation, and significant statistical difference (*p* < 0.05). The size and shape of the seeds are defined by the length, width, and perimeter, and the thickness and surface area that are calculated from the first two. The chan seeds from Jalisco had the lowest length, width, perimeter, and surface area, 3.275 mm, 2.443 mm, 10.139 mm, and 20.307 mm^2^, respectively. Seeds from Nayarit exhibited the greatest mean length (3.559 mm), which was significantly higher than that of seeds from Colima and Jalisco (*p* < 0.05). These lengths are similar to those reported for fenugreek seeds (Agrawal et al. 2015), and are smaller than those obtained for species of European spindle tree (Kaliniewicz et al. 2021). For the width, there is a significant difference between the seeds studied and that of Colima, presenting the maximum value (2.635 mm). The width of these seeds is comparable to those reported for certain species of *Viburnum* (Kaliniewicz and Choszcz 2021). The length and width of the chan seeds studied are like values reported for fenugreek seeds and pearl millet (Agrawal et al. 2015; Ramashia et al. 2018). For flaxseeds, the length is greater, and the width coincides with the values obtained for the chan seeds studied (Coşkuner and Karababa 2007). The largest perimeter corresponded to the Nayarit seeds with 12.202 mm and presents a significant statistical difference with respect to the other seeds.

**TABLE 1:** Physical parameters of chan seed ( *Hyptis suaveolens* ) from different geographical regions of Mexico.

	Colima	Jalisco	Nayarit
Length^†^ (mm)	3.346^b^ ± 0.314	3.275^b^ ±0.365	3.559^a^ ± 0.340
Width^†^ (mm)	2.635^a^ ± 0.243	2.443^c^ ± 0.258	2.552^b^ ± 0.238
Perimeter^†^ (mm)	10.782^b^ ± 0.865	10.139^c^ ± 1.087	12.202^a^ ± 1.089
Weight of 1000 seeds^†^ (g)	5.651^a^ ± 0.020	5.161^b^ ± 0.020	5.546^a^ ± 0.038
Volume by displacement* (mm^3^)	5.159^a^ ± 0.191	4.913^a^ ± 0.218	5.174^a^ ± 0.327
True density* (g/cm^3^)	1.088^a^ ± 0.012	1.056^b^ ± 0.002	1.060^b^ ± 0.012
Bulk density * (g/cm^3^)	0.616^a^ ± 0.010	0.621^a^ ± 0.012	0.620^a^ ± 0.012
Static angle of repose* (°)	28.580^b^ ± 0.340	28.310^b^ ± 0.350	29.730^a^ ± 0.350
Dynamic angle of repose* (°)	35.210^c^ ± 0.520	36.720^b^ ± 0.220	40.580^a^ ± 0.390

*Note:* Different letters indicate minimum significant difference, *p* < 0.05.

* Determinations made by sextuplicate.

^†^ Determinations result of 100 repetitions.

**TABLE 2:** Calculated physical parameters of chan seed ( *Hyptis suaveolens* ) from different geographical regions of Mexico.

	Colima	Jalisco	Nayarit
Surface area (mm^2^)	23.049^a^ ± 4.005	20.307^b^ ± 3.912	22.442^a^ ± 3.940
Thickness (mm)	1.181^b^ ± 0.109	1.249^a^ ± 0.132	1.147^b^ ± 0.107
Arithmetic diameter (mm)	2.358^ab^ ± 0.209	2.288^b^ ± 0.215	2.392^a^ ± 0.211
Geometrical diameter (mm)	2.128^a^ ± 0.189	2.091^a^ ± 0.198	2.130^a^ ± 0.189
Sphericity (%)	63.67^a^ ± 2.45	64.14^a^ ± 4.70	59.93^b^ ± 2.63
Porosity (%)	43.42^a^ ± 0.97	41.14^b^ ± 1.09	41.49^b^ ± 1.52
Volume PDI (mm^3^)	6.222^a^ ± 1.565	5.649^b^ ± 1.578	6.226^a^ ± 1.576
*V*_*d*_/*A*_*s*_ ratio	0.2238	0.2419	0.2305

*Note:* Determinations of 100 repetitions. Different letters indicate minimum significant difference, *p* < 0.05.

Abbreviations: *A*_*s*_, surface area; *V*_*d*_, volume per displacement; PDI, Procedure digitalization of images.

The weight of 1000 seeds (W_1000s_) of chan from Nayarit was 5.546 g, corresponding to the highest value with a significant difference from the other seeds. The values obtained are comparable to those reported for flaxseeds (Coşkuner and Karababa 2007; Krajewska et al. 2016). W_1000_ is used for the design of aeration, transport, drying, and storage equipment (Adubofuor et al. 2021).

The volume of a seed refers to the three‐dimensional space it occupies; it depends on the surface area, its linear dimensions, moisture, and temperature (Adubofuor et al. 2021). It is considered a quality attribute in the food industry and related to other quality parameters, such as the inverse relationship with texture (Sahin and Sumnu 2006). The smallest volume determined by displacement and corresponded to the Jalisco seeds with 4.913 mm^3^ and 5.649 mm^3^, respectively. In all cases, the PDI volume values were higher than those estimated by displacement, and a significant difference was found between seeds.

The dynamic angle of repose of a seed is always higher than the static angle of repose because the former is the angle with the horizontal at which the seed can no longer remain stacked and begins to slide; on the other hand, the static angle of repose refers to the angle at which the seed can remain stacked (Sahin and Sumnu 2006). Those angles of Nayarit seeds had the highest value and presented a significant statistical difference between the seeds from Colima and Jalisco. The static angle of repose varied from 28.31° to 29.73°. In previous reports, they found similar values for quinoa seeds (Rosa et al. 2020) and flaxseeds (Krajewska et al. 2016). The dynamic angle of repose was from 35.21° to 40.58°, which are similar values for flaxseeds (Krajewska et al. 2016).

Regarding the surface area and thickness, the seeds from Jalisco are the only ones that presented a significant statistical difference compared to the others, and the thickness of these seeds was the highest value (1.147 mm). This parameter is higher than reported for flaxseeds (Coşkuner and Karababa 2007). The seeds from Colima presented the largest surface area (23.049 mm^2^). The parameters that define the size of the seeds are important in the separation of foreign materials, to evaluate the quality of the materials; in fluid flow, mass, and heat transfer calculations (Sahin and Sumnu 2006). The relation between volume and the superficial area allows us to set up how the heat transfer behaves; as this ratio decreases, heat transfer through the seeds is favored (Adubofuor et al. 2021), as in the case of chan seeds where this ratio varied from 0.2238 to 0.2419.

The arithmetic and geometric diameters of the seeds depend on the dimensions of the seeds (Adubofuor et al. 2021). For the Nayarit seeds, the highest values were determined for the arithmetic (2.3692 mm) and geometric (2.130 mm) diameter; however, no significant difference was found between seeds. The values reported here are similar to those for flaxseeds (Coşkuner and Karababa 2007). The geometric diameter is used to estimate the projected area of seeds moving in a turbulent region of an air current, which determines the movement pattern of the seeds and the ease of removing foreign materials during cleaning by pneumatic means. The arithmetic diameter is used to design sieving and grading machines (Adubofuor et al. 2021).

The sphericity varied from 59.93% to 64.14%. The minimum value corresponded to the seeds of Nayarit, with a significant difference compared to the other seeds. These values are higher than those reported by Kaliniewicz and Choszcz (2021) for different species of *Viburnum*. The determined porosity varied from 41.14% to 43.42%. Colima seeds presented the highest porosity, with a statistically significant difference compared to the rest of the seeds in the study. The porosity of chan seeds is approximately 11% greater than that of quinoa seeds (Jan et al. 2019).

### 3.2 Composition of Chan Seeds

The composition of the chan seeds from Colima, Jalisco, and Nayarit is presented in Table 3. The moisture ranged from 6.81% to 7.63%. The lowest value corresponded to the Jalisco seeds, with a statistically significant difference concerning the seeds from Colima and Nayarit. In the Colima seeds, the maximum ash value (3.77%) and the lowest for the Nayarit seeds (3.56%) were determined, having a significant difference with respect to the others. The protein content is in an interval from 17.92% to 19.63%. The maximum value was registered for the seeds of Nayarit, with a statistically significant difference. The values obtained are like those reported for chia, canola, flaxseed, soybean, and sunflower seeds (Mondor 2023). Regarding the fat content, no significant statistical difference was found, and it varied from 17.81% to 18.38%. These results are similar to those reported by Mondor (2023) for soybean seeds. The crude fiber of the Nayarit seeds was 18.79% up to 20.85% for Colima seeds with a significant statistical difference between seeds. These values are like those reported for chia and sunflower seeds (Mondor 2023). The nitrogen‐free extract was calculated by difference obtaining values from 31.35% to 33.77% with a significant difference between the evaluated seeds. The physicochemical composition determined for the three seeds studied is like those reported for chan seeds (Morales‐Tovar et al. 2020).

**TABLE 3:** Composition of chan seed ( *Hyptis suaveolens* ) from different geographical regions of Mexico.

Component (%)	Colima	Jalisco	Nayarit
Moisture	7.46^a^ ± 0.10	6.81^b^ ± 0.03	7.63^a^ ± 0.22
Ash	3.77^a^ ± 0.03	3.71^a^ ± 0.03	3.56^b^ ± 0.03
Protein	17.92^b^ ± 0.29	18.02^b^ ± 0.21	19.63^a^ ± 0.12
Fat	18.38^a^ ± 0.13	18.22^ab^ ± 0.25	17.81^b^ ± 0.25
Crude fiber	20.85^a^ ± 0.16	19.46^b^ ± 0.23	18.79^c^ ± 0.20
NFE	31.35^c^ ± 0.77	33.77^a^ ± 0.14	32.58^b^ ± 0.23

*Note:* The analysis was carried out in triplicate. Different letters indicate minimum significant difference, *p* < 0.05, NFE was calculated via the difference.

Abbreviation: NFE, Nitrogen‐free extract.

### 3.3 Fatty Acid Composition

The fatty acid composition of the chan seeds is shown in Table 4. A predominance of linoleic acid (ῳ‐6), which is a polyunsaturated fatty acid, is observed in a range of 74.67%–78.19%, only comparable with the oil of safflower and superior to corn, soybean, flaxseed, chia, and sunflower oils among others (Fernandes et al. 2021; Kostik et al. 2013; Zamani Ghaleshahi et al. 2020), and oleic acid (ῳ‐9) (monounsaturated fatty acid), with values from 9.31% to 11.32%. Saturated fatty acids (SFAs) vary from 12.51% to 14.62%, with a predominance of palmitic acid. The SFA content of these oils is comparable to that of olive and soybean oils (Kostik et al. 2013; Mondor 2023). The high content of linoleic and oleic acids makes chan seeds oil of interest for food use. Linoleic acid is essential because humans cannot synthesize it. It has a fundamental role in growth; increases high‐density cholesterol (HDL); decreases low‐density cholesterol (LDL); and helps prevent cardiovascular diseases, diabetes, arthritis, and autoimmune diseases, among others (Fernandes et al. 2021; Kostik et al. 2013). For its part, oleic acid lowers LDL without affecting HDL levels (Kostik et al. 2013). The linoleic acid content of the chan seeds is higher than that reported for olive, sunflower, soybean, corn, and canola oil seeds, and similar content with safflower seeds oil (Kostik et al. 2013). The PUFA/SFA ratio is associated with human health; therefore, increasing its value favors the reduction of serum cholesterol and atherosclerosis; at the same time, cardiovascular diseases can be prevented. The PUFA/SFA ratio of the chan seed oils studied ranged from 5.12 to 6.25. These values are comparable to those found for goji, sunflower, and safflower seed oils and higher than those reported for canola, peanut, olive, corn, and cotton oils, among others (Fernandes et al. 2021; Kostik et al. 2013; Zhang et al. 2022).

**TABLE 4:** Fatty acid composition of chan seed ( *Hyptis suaveolens* ) from different geographical regions of Mexico.

Fatty acids (%)	Colima	Jalisco	Nayarit
Palmiic acid (16:0)	11.12^a^ ± 0.34	11.69^a^ ± 0.11	9.96^b^ ± 0.25
Stearic acid (18:0)	2.90^a^ ± 0.04	2.93^a^ ± 0.04	2.55^b^ ± 0.00
Oleic acid (18:1) (ῳ‐9)	11.32^a^ ± 0.39	10.58^a^ ± 0.03	9.31^b^ ± 0.56
Linoleic acid (18:2) (ῳ‐6)	74.67^b^ ± 0.09	74.79^b^ ± 0.13	78.19^a^ ± 0.19
Total SFA	14.02^a^ ± 0.30	14.62^a^ ± 0.15	12.51^b^ ± 0.25
Total MUFA	11.32^a^ ± 0.39	10.58^a^ ± 0.03	9.31^b^ ± 0.56
Total PUFA	74.67^b^ ± 0.09	74.79^b^ ± 0.13	78.19^a^ ± 0.19
PUFA/SFA	5.33^b^ ± 0.11	5.12^b^ ± 0.06	6.25^a^ ± 0.14

*Note:* The analysis was carried out in duplicate. Different letters indicate minimum significant difference, *p* < 0.05.

Abbreviations: MUFA, monounsaturated fatty acids; PUFA, polyunsaturated fatty acids; SFA, saturated fatty acids.

### 3.4 Correlation Between Physical Properties of Chan Seeds

The Pearson correlation coefficient is an index that allows measuring the level of linear association between parameters, whose scale ranges from −1 to +1. Their relationship can be direct or inverse. In the first case, the variation of both properties is in the same direction. In the inverse relationship, when one of the properties increases, the other decreases. As a correlation's absolute value increases, the association level increases (Schober and Schwarte 2018). The correlation between the physical properties of the chan seeds studied is presented in Table 5. One hundred and twenty pairs of comparisons were made with a confidence level of 95%. Of these, 50 are correlations greater than 0.8. A perfect and positive correlation was determined between the geometric diameter (*D*_*g*_) and the volume per displacement (*V*_*d*_); between the porosity (Ԑ) and the true density (ρ_t_); and between the surface area (*A*_*s*_) and W_1000s_. The arithmetic diameter (*D*_*a*_) and the thickness (*E*) present a perfect inverse correlation; that is, as one of these increases, the other decreases. The parameters that correlate with more than 0.8 and with the largest number are *E* and *D*_*a*_. It should be noted that most of the correlations of the thickness with the different parameters evaluated are inverse; that is, the less thick chan seeds have a greater length, perimeter, weight, volume, *A*_*s*_, *D*_*a*_, *D*_*g*_, and static angle of repose (Ɵ_e_).

**TABLE 5:** Coefficients of linear correlation between physical properties of seeds of chan.

	*L*	*W*	*P*	*P*_1000s_	*V*_*d*_	*V*_PDI_	*δ*_ab_	*δ*_vt_	*A*_*s*_	*E*	*D*_*a*_	*D*_*g*_	φ	Ԑ	Ɵ_*e*_
*W*	0.315														
*P*	**0.998**	0.378													
*P*_100s_	0.532	**0.971**	0.588												
*V*_*d*_	0.729	**0.879**	0.773	**0.967**											
*V*_PDI_	0.698	**0.900**	0.744	**0.978**	**0.999**										
δ_ab_	0.091	**−0.917**	0.024	−0.795	−0.615	−0.650									
δ_vt_	−0.165	**0.884**	−0.099	0.747	0.554	0.591	**−0.997**								
*A*_*s*_	0.526	**0.973**	0.582	**1.000**	**0.965**	**0.976**	−0.799	0.752							
*E*	**−0.891**	−0.712	**−0.919**	**−0.859**	**−0.962**	**−0.947**	0.371	−0.300	**−0.855**						
*D*_*a*_	**0.888**	0.717	**0.917**	**0.862**	**0.962**	**0.949**	−0.378	0.307	**0.858**	**−1**					
*D*_*g*_	0.725	**0.882**	0.770	**0.969**	**1.000**	**0.999**	−0.620	0.559	**0.967**	**−0.959**	**0.961**				
φ	**−0.990**	−0.179	**−0.979**	−0.408	−0.626	−0.591	−0.229	0.302	−0.402	**0.819**	**−0.815**	−0.622			
Ԑ	−0.134	**0.898**	−0.068	0.767	0.580	0.616	**−0.999**	**1.000**	0.772	−0.309	0.337	0.585	0.272		
Ɵ_e_	**0.998**	0.255	**0.992**	0.478	0.685	0.652	0.153	−0.227	0.472	**−0.861**	**0.857**	0.681	**−0.997**	−0.196	
Ɵ_*d*_	**0.868**	−0.198	**0.833**	0.041	0.293	0.249	0.574	−0.634	0.034	−0.548	0.542	0.287	**−0.929**	−0.609	**0.897**

*Note:* Negative values correspond to inverse correlations.

Abbreviations: *L*, length; *W*, width; *P*, perimeter; *P*_1000s_, thousand seed weight; δ_ab_, bulk density; δ_at_, true density; *V*_*d*_, volume per displacement; *V*_PDI_, volume per procedure digitalization of images; *E*, thickness; *D*_*a*_, arithmetic diameter; *D*_*g*_, geometric diameter; *A*_*s*_, surface area; φ, sphericity; Ԑ, porosity; Ɵ_e_, static angle of repose; Ɵ_d_, dynamic angle of repose.

The thickness is correlated directly with the sphericity. Unlike thickness, the correlations of D_a_ with these parameters are direct, which implies that as *D*_*a*_ increases, these also increase. Additional correlations without significant variation were observed between perimeter and porosity, bulk density, and ρ_t_. Similarly, associations were identified between seed length and ρ_b_, as well as between the dynamic angle of repose (θ_d_) and both W_1000s_ and A_s_. between the length and ρ_b_; and between the dynamic angle of repo Regarding the two different ways of determining the volume of chan seeds, no difference is observed between them since they maintain a good correlation (0.999). Seed length and width have been considered fundamental criteria for postharvest seeds handling (Kaliniewicz and Choszcz 2021). Both parameters of the chan seeds correlate to a greater or lesser extent with the other parameters evaluated. Previous studies with seeds of the genus *Viburnum* emphasize the importance of width as a criterion separation. For seeds of the genus *Fagus*, the length was considered the most important parameter to classify them (Kaliniewicz and Choszcz 2021).

Furthermore, there is a strong correlation between some physical properties of chan seeds and saturated, monounsaturated, and polyunsaturated fatty acids (Table 6). SFA and MUFA present a very strong inverse correlation with the dimensional parameters L and P, as well as with the dynamic and static angles of repose. A very strong direct correlation exists between sphericity, thickness, and SFA, and in the case of MUFA, with φ. It is important to emphasize that the correlation between these properties and PUFA is contrary to that determined for SFA and MUFA (Table 6). Defining these correlations may allow for establishing a method for selecting chan seeds according to their quality through these physical properties. Furthermore, in this case, the origin of the seeds is a determining factor in quality. The highest PUFA content was determined in seeds from Nayarit (Table 4), which directly correlates with the highest values of *L*, *P*, Ɵ_e_, and Ɵ_d_ (Tables 1, 2).

**TABLE 6:** Coefficients of linear correlation between physical properties and fatty acids of seeds of chan.

	L	P	Ɵ_e_	Ɵ_d_	E	φ
SFA	−0.999	−1.000	−0.995	−0.849	0.907	0.984
MUFA	−0.817	−0.776	−0.851	−0.995	*	0.889
PUFA	0.963	0.943	0.978	0.970	−0.736	−0.991

Abbreviations: L, length; *P*, perimeter; Ɵe, static angle of repose; Ɵ_d_, dynamic angle of repose; E, thickness; φ, sphericity; SFA, saturated fatty acids; MUFA, monounsaturated fatty acids; PUFA, polyunsaturated fatty acids; *, moderate correlation.

### 3.5 Surface Microstructure of Chan Seeds

**FIGURE 1:**
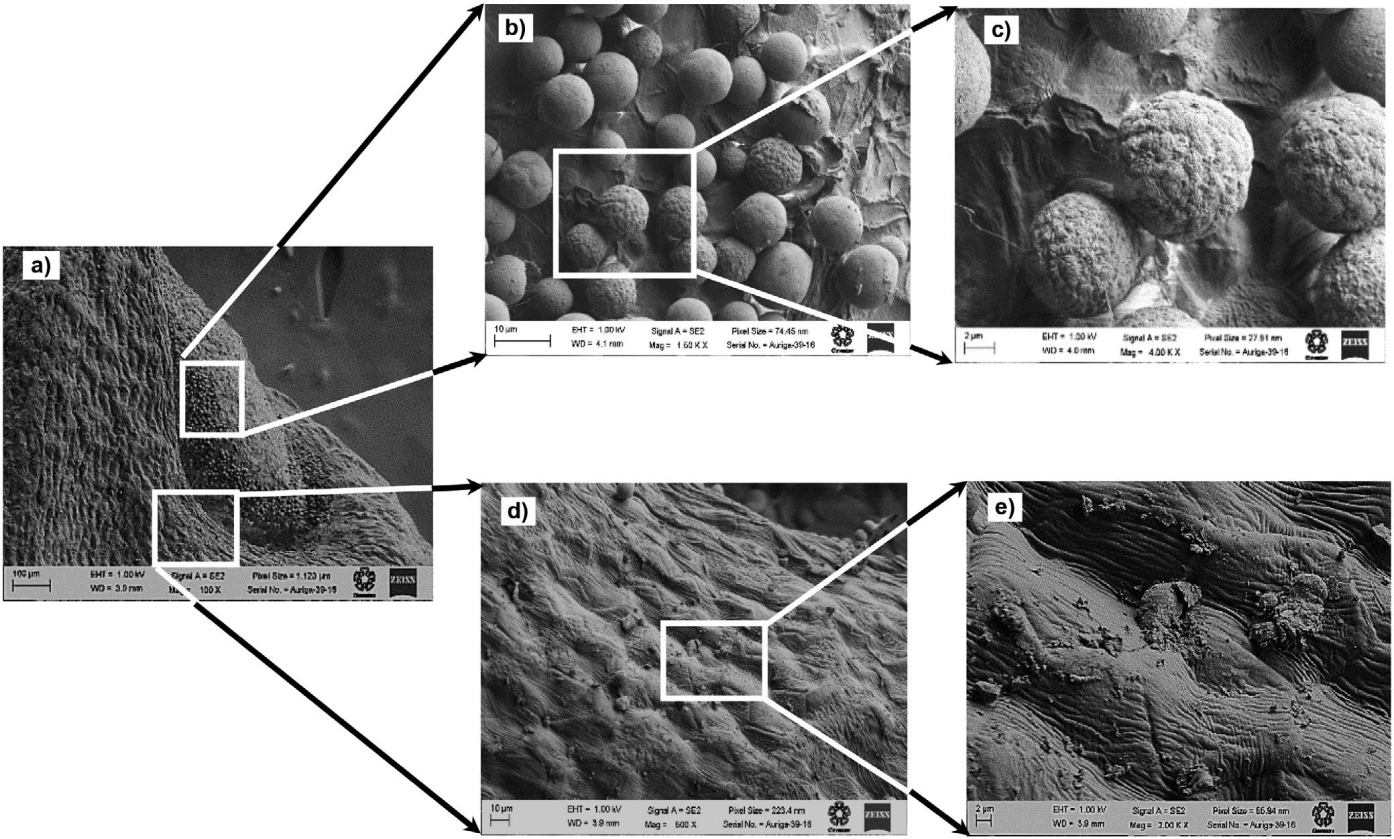
Scanning electron micrographs of different sections of the distal side of the pericarp of chan ( *Hyptis suaveolens* ) seed from the crop of Colima. (a) Myxocarp, (b) and (c) globular zone or granular; (d) and (e) angular morphology.

**FIGURE 2:**
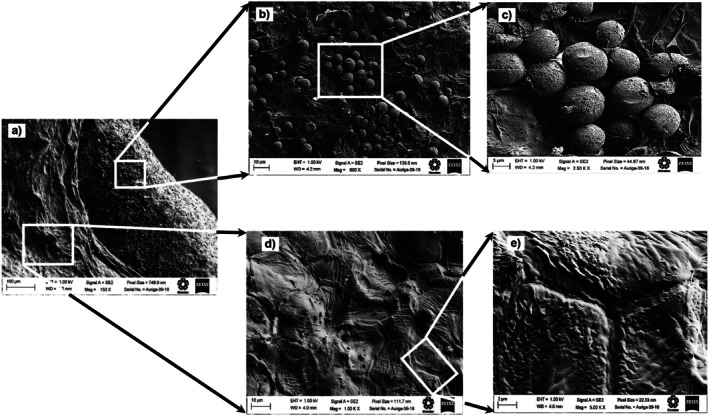
Scanning electron micrographs of different sections of the distal side of the pericarp of chan ( *Hyptis suaveolens* ) seed from the crop of Jalisco. (a) myxocarp, (b) and (c) globular zone or granular; (d) and (e) angular morphology.

**FIGURE 3:**
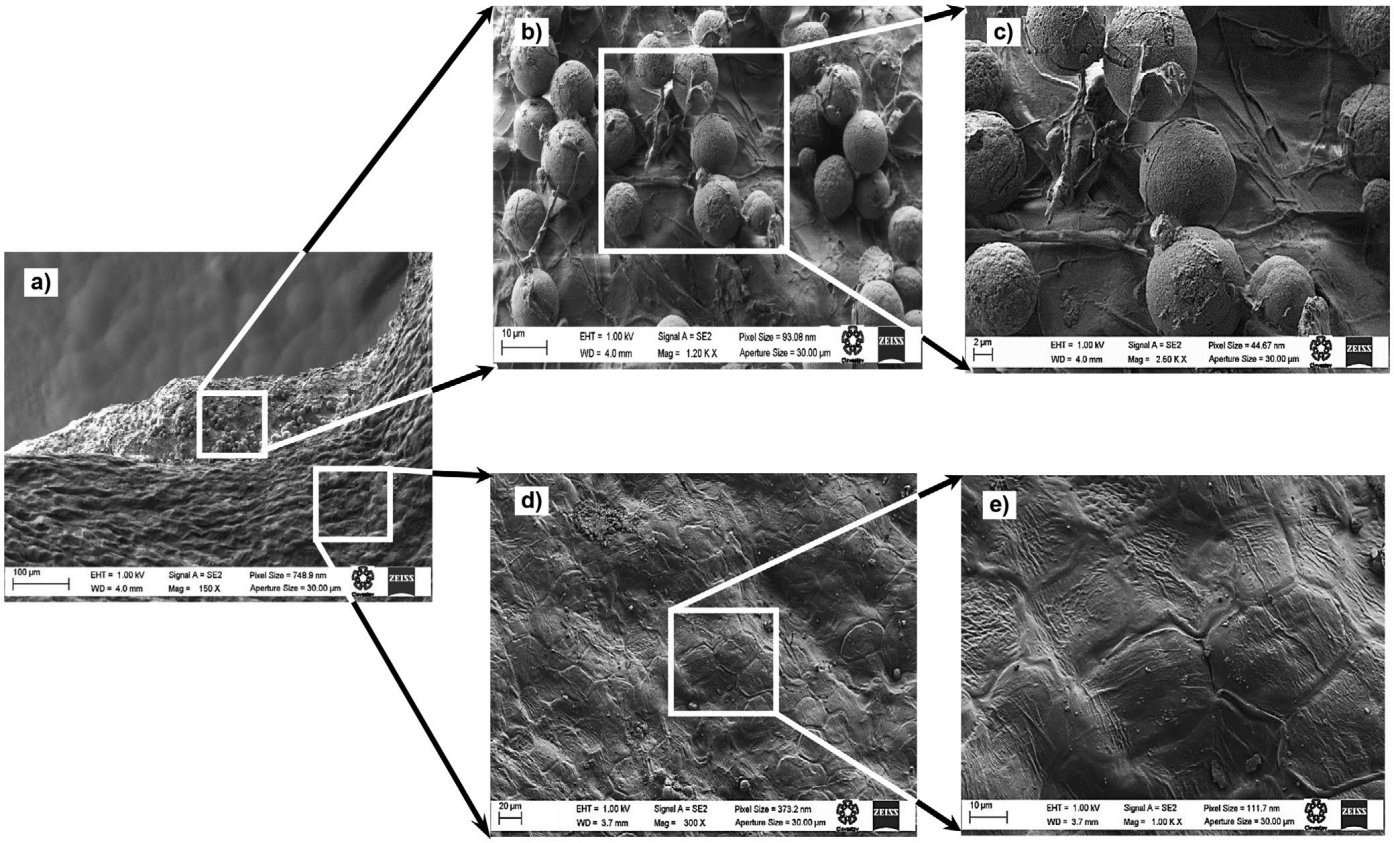
Scanning electron micrographs of different sections of the distal side of the pericarp of chan ( *Hyptis suaveolens* ) seed from the crop of Nayarit. (a) myxocarp, (b) and (c) globular zone or granular; (d) and (e) angular morphology.

The surface microstructure of chan seeds was studied using scanning electron microscopy (SEM) and atomic force microscopy (AFM). Figures 1, 2, 3 show the micrographs obtained by SEM of the distal end of the chan seeds. In those, the similarity of the structure of the epicarp of the three different chan seeds is observed. In all cases, two predominant areas can be distinguished. In the basal region of the seed, the distal area stands out for its surface bilobed areola with papillary structure (Figures 1a, 2a, 3a) (Ryding 1992). Besides, on most of the surface of the exocarp, intermixed mucilaginous and non‐mucilaginous cells are distinguished (Figures 1d,e, 2d,e, 3d,e). This type of structural conformation is known as mixocarpy (Kreitschitz and Gorb 2018; Moon et al. 2009). This is a characteristic of seeds and nuts of the *Lamiaceae* family. These cells are distinguished by being isodiametric, angular, and slightly elongated, according to what was previously reported for nuts from the *Lamiaceae* family. In *Hyptis Suaveolens* , cells have been observed to elongate up to 100 times, measure up to 2 mm, and produce a large amount of mucilage (Ryding 1992). The ability to form mucilage allows the seed to adapt to dry or disturbed habitats and has an important role in adhesion to the soil and the regulation of germination (Kreitschitz and Gorb 2018). Chan seeds are typically dark brown, flattened, and slightly rough, with an emarginate apex and a V‐shaped or subtly notched base.

**FIGURE 4:**
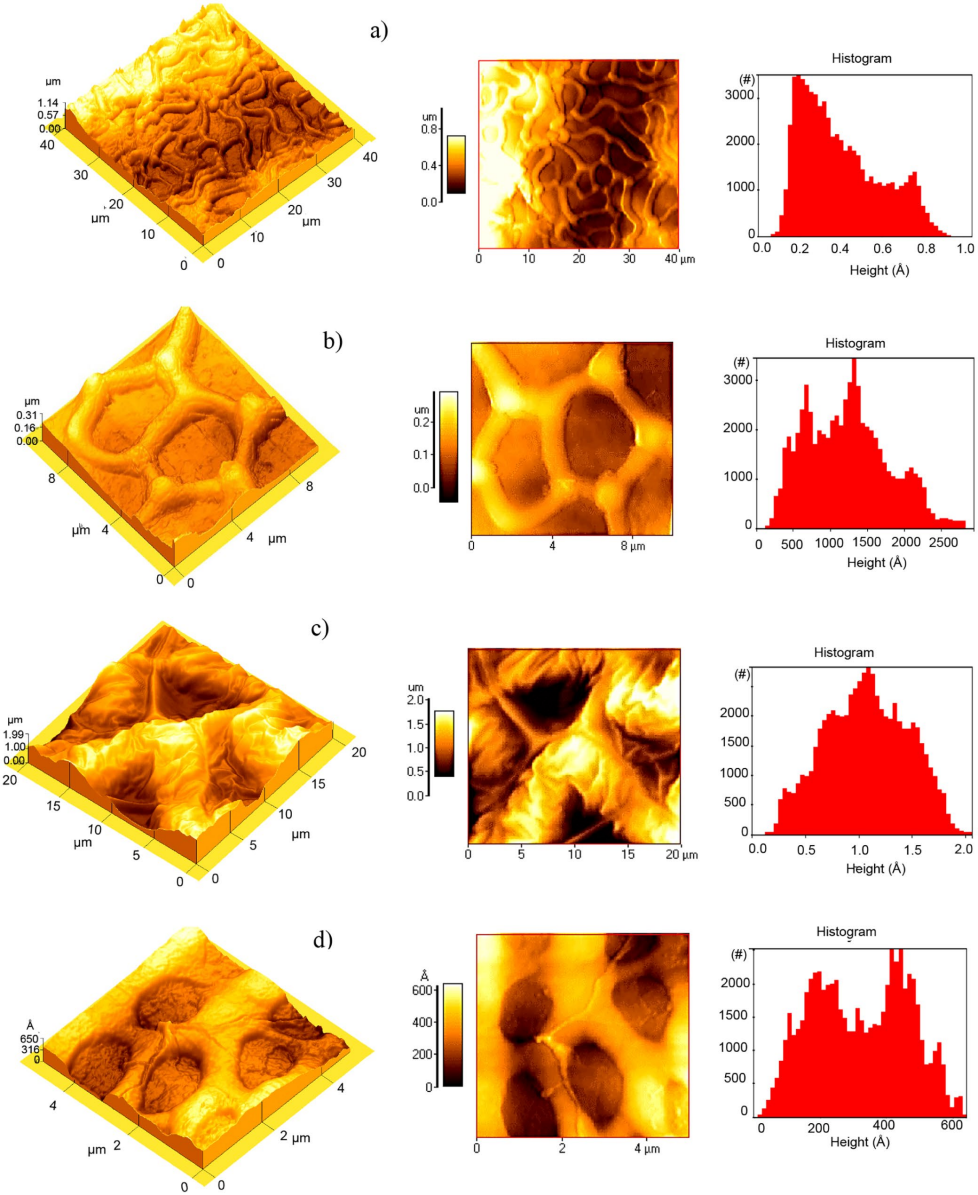
Atomic force micrographs of the surface of chan ( *Hyptis suaveolens* ) seed. Exocarp: (a) elongated mucilaginous cells and (b) quasi‐isodiametric angular cells; endocarp: (c) angular zone and (d) lobular zone or granular.

AFM micrographs show the 3D topography of the two predominant regions of the chan seed pericarp (Figure 4). In Figure 4a, an area of 40 × 40 μm was scanned, where the slightly elongated isodiametric cells are observed, which are located near the base of the seed and have a roughness of 0.164 μm and an average height of 0.356 μm (Table 7). A close‐up of this zone (Figure 4b) shows isodiametric and angular cells, which represent most of the seed surface, with an average elevation of 0.124 μm in an area of 12.31 μm^2^ (Table 7). Figure 4c stands for a 441.5‐μm area of the internal part of a section of the endocarp, in which the edges of the angular cells are observed with an average height of 1.070 μm and roughness of 0.320 μm (Table 7). Finally, an internal image of the areola is presented, where the lobular zone with an average height of 0.0322 μm is observed (Table 7). This type of micromorphology has already been observed in other nuts of the *Lamiaceae* family (Moon et al. 2009).

**TABLE 7:** AFM micrograph parameters of the different sections of the chan seed.

	Section*			
	a	b	c	d
Average roughness (μm)	0.164	0.0562	0.320	0.0125
Average height (μm)	0.356	0.124	1.070	0.0322
Surface área (μm^2^)	1623	100.2	441.5	24.950
Volume (μm^3^)	641.4	12.31	428.1	0.8012

* According to AFM micrograph.

## 4 Conclusions

The physical properties of chan seeds are an important tool that can contribute to the development of post‐harvest operations and technologies that allow for intensive cultivation, management, industrialization, and large‐scale distribution in the near future. The values found for the (*V*_*d*_/*A*_*s*_) ratio favor heat transfer through the seed. One hundred and twenty comparisons between pairs of parameters were made; 40.8% correspond to a linear correlation greater than 0.8 (*p* < 0.05). Seed selection by the parameters *L*, *P*, φ, and E can be a good indicator of seed quality based on the content of SFA, MUFA, and PUFA. Its protein content is comparable to that of pseudocereals. Linoleic acid (ῳ‐6) predominates, which is essential for growth, cholesterol balance, and the prevention of various diseases. Chan seed oil is of high nutritional quality. It can be considered as a prebiotic due to its high crude fiber content. In the microstructure of the seeds, the presence of myxocarp stands out, which is characterized by intermingled mucilaginous and non‐mucilaginous cells.

## Author Contributions

**Juan Alfredo Salazar‐Montoya:** conceptualization (equal), data curation (equal), formal analysis (equal), investigation (equal), methodology (equal), validation (equal), writing – review and editing (equal). **María Dolores Díaz‐Cervantes:** formal analysis (equal), investigation (equal), methodology (equal), writing – original draft (equal). **Emma Gloria Ramos‐Ramírez:** supervision (equal), validation (equal), writing – review and editing (equal).

## Funding

No funding was obtained for this study. This research did not receive any specific grant from funding agencies in the public, commercial, or non‐profit sectors.

## Conflicts of Interest

The authors declare no conflicts of interest.

## Data Availability

Data will be made available on request.
